# In-Depth Study of Low-Complexity Domains: From Structural Diversity to Disease Mechanisms

**DOI:** 10.3390/cells14221752

**Published:** 2025-11-09

**Authors:** Haixia Xu, Kaili Zhou, Lianren Xia, Kejin Ren, Yongjie Xu

**Affiliations:** 1 College of Life Science, Xinyang Normal University, Xinyang 464000, China; hxxu@xynu.edu.cn (H.X.); zhoukaili@xynu.edn.cn (K.Z.); xialianren@xynu.edn.cn (L.X.); kejin@xynu.edn.cn (K.R.); 2 Institute for Conservation and Utilization of Agro-Bioresources in Dabie Mountain, Xinyang Normal University, Xinyang 464000, China

**Keywords:** LCDs, LLPS, biomolecular condensates, neurodegenerative diseases, cancer, structural biology, targeted therapy

## Abstract

Low-complexity domains (LCDs) are protein regions characterized by a simple amino acid composition and low sequence complexity, as they are typically composed of repeats or a limited set of a few amino acids. Historically dismissed as “garbage sequences”, these regions are now acknowledged as critical functional elements. This review systematically explores the structural characteristics, biological functions, pathological roles, and research methodologies associated with LCDs. Structurally, LCDs are marked by intrinsic disorder and conformational dynamics, with their amino acid composition (e.g., G/Y-rich, Q-rich, S/R-rich, P-rich) dictating structural tendencies (e.g., β-sheet formation, phase separation ability). Functionally, LCDs mediate protein–protein interactions, drive liquid–liquid phase separation (LLPS) to form biomolecular condensates, and play roles in signal transduction, transcriptional regulation, cytoskeletal organization, and nuclear pore transportation. Pathologically, LCD dysfunction—such as aberrant phase separation or aggregation—is implicated in neurodegenerative diseases (e.g., ALS, AD), cancer (e.g., Ewing sarcoma), and prion diseases. We also summarize the methodological advances in LCD research, including biochemical (CD, NMR), structural (cryo-EM, HDX-MS), cellular (fluorescence microscopy), and computational (MD simulations, AI prediction) approaches. Finally, we highlight current challenges (e.g., structural heterogeneity, causal ambiguity of phase separation) and future directions (e.g., single-molecule techniques, AI-driven LCD design, targeted therapies). This review provides a comprehensive perspective on LCDs, illuminating their pivotal roles in cellular physiology and disease, and offering insights for future research and therapeutic development.

## 1. Introduction

Proteins are the primary executors of cellular functions, a role predominantly determined by their specific three-dimensional structures. This paradigm, however, is expanded and complicated by the presence of regions characterized by a simple amino acid composition and low sequence complexity (i.e., they are dominated by one or a few amino acid types and lack diversity), known as low-complexity domains (LCDs)*,* which often lack a stable three-dimensional structure yet fulfill critical functions [1,2]. These LCDs are continuous segments within protein sequences, frequently composed of repeats or simple patterns of only a limited number of amino acids, and are defined as regions comprising at least 20 amino acids, with 40% or more of the sequence consisting of a single type of amino acid [3]. Although traditionally regarded as non-functional “garbage sequences”, LCDs are now acknowledged for their essential roles and are ubiquitously distributed across life, with particularly high prevalence in eukaryotic organisms.

The functional diversity of LCDs is reflected in their classification into over 400 types based on their predominant amino acid composition, each with distinct functional preferences [3]. Composition varies greatly; some LCDs are enriched in polar residues like glutamine (Q) and asparagine (N), others in aromatic amino acids like tyrosine (Y) and phenylalanine (F), and still others in small-side-chain amino acids like glycine (G) and alanine (A). This compositional diversity directly dictates their structural properties and functional mechanisms. For example, the clinical relevance of this classification is evident in neurodegenerative diseases, where polyQ-rich LCDs are pathogenic drivers; in Huntington’s disease, an abnormal expansion of the polyQ tract in the huntingtin protein leads to its dysfunction [4].

Breakthroughs in understanding LCDs’ function have been driven recently by the theory of liquid–liquid phase separation (LLPS) [5]. LLPS is a physical process whereby biomolecules (e.g., proteins, RNA) demix from a homogeneous solution into dense, liquid-like droplets surrounded by a dilute phase, much like oil separating in water [6]. These droplets, known as biomolecular condensates, function as membraneless organelles that compartmentalize and regulate fundamental cellular processes. LCDs are now understood as key drivers of biomolecular condensates, which form membraneless organelles that compartmentalize critical processes like transcriptional regulation and RNA processing [6,7]. Consequently, LCD dysfunction is implicated in serious diseases, including amyotrophic lateral sclerosis (ALS), frontotemporal dementia (FTD), and various cancers [8].

This article will systematically explore the structural characteristics, functional mechanisms, and pathological roles of LCDs. By integrating and critically evaluating recent research, we aim to provide a comprehensive perspective on how the unique properties of LCDs underpin their vital cellular functions and how their dysregulation leads to disease, thereby illuminating new targets and strategies for future therapeutic development.

## 2. Structural Characteristics and Classification of LCDs

### 2.1. Sequence Composition and Structural Diversity

**Figure 1:**
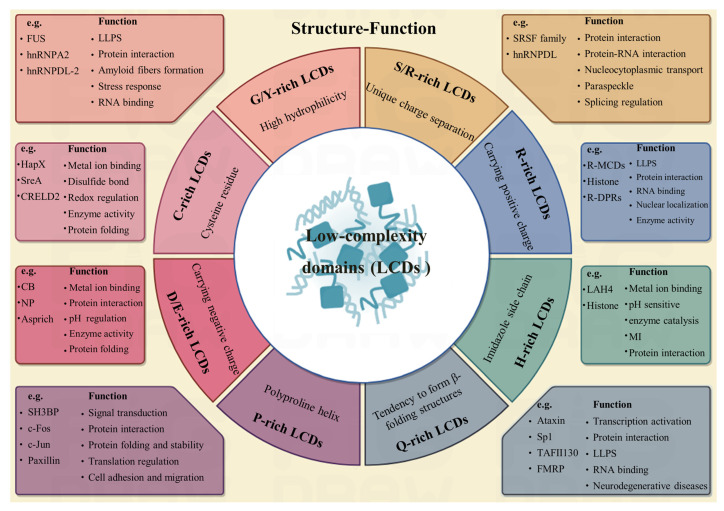
Structural characteristics and biological functions of LCDs. The central module showing eight representative LCD types classified by amino acid composition (G/Y-rich, S/R-rich, R-rich, H-rich, Q-rich, P-rich, D/E-rich, C-rich) and their structural tendencies. Right and left branches illustrate the typical protein with LCDs structure and LCDs-mediated biological processes, including LLPS-driven biomolecular condensates [5], protein interaction [14], stress response [15], RNA binding [3], metal ion binding [3], redox regulation [16], enzyme activity regulation [17], protein folding and stability [12], translation regulation [3], cell adhesion and migration [18], nucleocytoplasmic transport [19], splicing regulation and signal transduction [20]. MI, Membrane interaction; CB, calbindin; NP, nucleolar protein.

The most fundamental feature of LCDs is the simplicity and repeatability of their amino acid composition, typically dominated by one or two amino acids that constitute a high proportion (usually ≥40%) of the sequence [3,9]. The compositional simplicity of low-complexity domains (LCDs) facilitates their classification into functionally distinct categories, each characterized by specific amino acid enrichment and corresponding structural and functional properties (Figure 1). For instance, S/R-rich LCDs are enriched in serine (S) and arginine (R) (totaling ≥40%), exhibiting unique charge separation and high hydrophilicity, which are primarily implicated in RNA binding and splicing regulation [10]. G/Y-rich LCDs, characterized by a predominance of glycine (G) and tyrosine (Y) (totaling ≥40%), display a weak propensity for β-sheet formation and a strong capacity for phase separation, often contributing to the formation of biomolecular condensates [9]. P-rich LCDs, with a proline (P) content of ≥30%, typically adopt polyproline helix structures and are primarily involved in signal transduction and cell adhesion [2,9]. Q-rich LCDs, with a glutamine (Q) content of ≥35%, remain intrinsically disordered or form gel-like structures, playing critical roles in transcriptional regulation and aggregation related to neurodegenerative diseases [2,11]. C-rich LCDs, characterized by a cysteine (C) content of ≥15%, are capable of forming disulfide bonds and function in redox regulation and metal ion binding [3]. Lastly, H-rich LCDs, with a histidine (H) content of ≥20%, are pH-sensitive due to their protonatable imidazole side chains, serving as molecular sensors for environmental cues [3,9].

The functional diversity of LCDs is reflected in their vast combinatorial potential. Strikingly, proteomic analyses indicate that all 400 possible di-amino-acid LCD types—where two amino acids dominate the composition—are represented in nature, albeit with varying abundance [3,12]. This prevalence underscores that LCDs are a fundamental and widespread feature of proteomes. Critically, this amino acid composition directly dictates their structural properties. For instance, LCDs enriched in polar uncharged amino acids like glutamine (Q) and asparagine (N) tend to remain intrinsically disordered, whereas those rich in aromatic residues like tyrosine (Y) and phenylalanine (F) have a greater propensity to form beta-sheet structures [2]. However, structural propensity is not solely determined by broad physicochemical categories; even LCDs composed of similar hydrophobic amino acids, such as valine (V) and leucine (L), can exhibit distinct secondary structural tendencies, indicating that specific sequence arrangement also plays a crucial role.

The structural diversity of LCDs is further demonstrated by their variability in length and sequence patterns. LCD length can vary significantly, from tens to hundreds of amino acid residues. For example, the ARID1A protein in mammalian SWI/SNF complexes contains an LCD of approximately 523 amino acids, whereas the LCD in the FUS protein is relatively short at about 173 residues [13]. Sequence patterns within LCDs also range from simple homorepeats (e.g., Q-rich) to more complex semi-repetitive motifs.

### 2.2. Structural Characteristics and Conformational Dynamics

Unlike traditional structured domains, a defining characteristic of LCDs is their intrinsic structural disorder and conformational heterogeneity [21]. While most LCDs lack a stable three-dimensional fold and exist as dynamic ensembles or form only transient structures in solution, their disorder is not absolute. Under specific conditions—such as changes in pH, temperature, or ionic strength, or upon binding to partner molecules—many LCDs can undergo transitions to more ordered states [2]. The propensity for such structural ordering varies among LCD types; for instance, those enriched in aromatic amino acids often form beta-sheet structures, while LCDs rich in small-side-chain amino acids like glycine (G) and alanine (A) tend to adopt more flexible conformations [21].

This conformational plasticity is fundamental to LCD function. The ability to transition dynamically between disordered and ordered states is often directly linked to specific biological activities. A key example occurs under cellular stress, where certain LCDs fold into beta-sheet structures to nucleate the assembly of stress granules [5]. This structural dynamism provides a mechanistic explanation for the involvement of LCDs in diverse processes, from transcriptional regulation and signal transduction to cytoskeleton organization [3].

### 2.3. LCDs and Intermediate Silk Proteins

**Figure 2:**
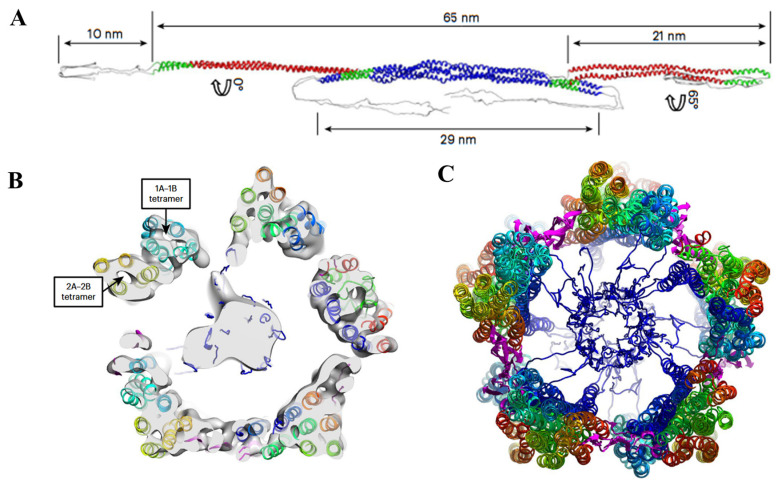
Structural Landscape of vimentin IFs featuring LCDs. (**A**). Structural model of a vimentin IFs tetramer. The α-helices proximal to the C terminus LCDs are in red (2A–2B dimers), and the α-helices closer to the N-terminus LCDs are in blue 1A–1B dimers. Regions of high conservation within the vimentin helices are depicted in green. The highly conserved regions of vimentin helices are shown in green. (**B**). Cross-sectional electron density map of Vimentin IFs, revealing all five protofibrils and the luminal fiber. (**C**). The cryo-EM cross-section structure of the Vimentin filament, demonstrating lateral interactions between protofibrils via the tail domains and central interactions through the head domains. The 2A–2B dimers significantly contribute to the outer surface configuration of vimentin IFs, while the 1A–1B dimers predominantly line the inner surface. Figures are adapted from Eibauer et al., 2024 [22].

The link between LCD composition and cellular function is powerfully illustrated by intermediate filament (IF) proteins, which are typically defined by their glycine-rich LCDs. IFs are essential components of the cytoskeleton, providing cells with mechanical support and structural stability. A defining feature of IFs proteins is their tripartite structure: a central alpha-helical rod domain flanked by N-terminal head and C-terminal tail domains [21]. These head and tail domains are typically LCDs, rich in glycine and other small-side-chain amino acids, and they are critical for IF assembly (Figure 2A). Recent research on vimentin, a key IF protein, has illuminated the precise structural role of this LCD. Using cryo-electron microscopy (cryo-EM) and tomography, Eibauer et al. revealed that the cross-section of a vimentin filament contains 40 α-helices organized into five protofibrils [22]. Intriguingly, the structurally dynamic head LCDs form a central filament core, while the tail LCDs mediate lateral connections between protofibrils (Figure 2B,C). This discovery demonstrates how LCDs work synergistically with well-folded helical domains to construct biopolymers with remarkable mechanical strength and elasticity.

Beyond their fundamental role in assembly, IF LCDs are involved in diverse cellular processes. For instance, they facilitate the reorganization of the IF network during cell division and can serve as platforms for protein interactions in cellular signaling pathways [21]. Furthermore, mutations or dysregulation of these domains are implicated in a spectrum of diseases, including skin disorders, neurodegenerative conditions, and cancer, underscoring their physiological importance.

### 2.4. Liquid Crystal Characteristics and Phase Separation Ability

Beyond structural roles, amino acid composition is the primary determinant of an LCD’s capacity for liquid–liquid phase separation (LLPS). Under appropriate conditions, many LCDs can undergo LLPS, a process analogous to oil-water separation but occurring at the molecular level [5]. During LLPS, proteins containing LCDs condense from a homogeneous solution into dense, protein-rich droplets suspended within a dilute, protein-poor phase. This phase separation behavior is governed by the amino acid composition and sequence patterns of the LCDs. Research indicates that aromatic residues (e.g., tyrosine, phenylalanine) and polar residues (e.g., serine, glycine, glutamine) play critical, distinct roles: aromatic amino acids often act as “stickers” that mediate specific inter-chain interactions, while polar residues serve as “spacers” that modulate the flexibility and dynamics of these interactions [23]. Furthermore, net charge and overall hydrophobicity significantly influence the phase separation threshold. Different types of LCDs exhibit distinct phase separation characteristics, reflecting their adaptation to specific cellular contexts. For instance, the LCD of the FUS protein undergoes phase separation at low salt concentrations, whereas the LCD of BRG1 requires higher protein concentrations or the presence of macromolecular crowding agents [13]. These differences suggest that various LCDs have evolved to function in distinct biological processes and under specific intracellular environmental conditions.

## 3. Biological Functions of LCDs

### 3.1. Protein Interactions and Formation of Multi Protein Complexes

One of the most fundamental biological functions of LCDs is to mediate protein–protein interactions and promote the formation of multi-protein complexes [24]. Their intrinsic disorder and structural flexibility allow LCDs to form transient yet specific interactions with diverse partners, establishing them as central hubs in intracellular interaction networks [5]. A hallmark of LCD-mediated interactions is their multivalent and dynamic nature. “Multivalency” refers to the ability of a single LCD to engage with multiple partners simultaneously, while “dynamics” describes the rapid association and dissociation of these contacts. This enables LCDs to flexibly modulate the composition and activity of protein complexes in response to cellular cues. For example, in transcriptional regulation, LCD-containing transcription factors can dynamically assemble complexes by concurrently interacting with multiple co-activators and RNA polymerase II [14,25].

The interaction capacity of LCDs is dictated by their amino acid composition. Aromatic residues, particularly tyrosine, are crucial for mediating LCD-LCD interactions via π-π stacking, which drives self-association and phase separation in proteins like EWS [26]. Additionally, electrostatic interactions between charged residues (e.g., arginine and aspartic acid) significantly contribute to LCD interaction networks [13]. Beyond self-association, LCDs can drive selective heterotypic interactions leading to co-condensation. A key example is the specific interaction between LCDs of FET family proteins (FUS, EWS, TAF15) and LCDs of various subunits in the mammalian SWI/SNF complex, resulting in functional co-condensates [13]. These specific heterotypic interactions are vital for processes like transcriptional regulation and chromatin remodeling, likely forming the basis for organizing intracellular functional modules.

### 3.2. LLPS and Formation of Biomolecule Aggregates

LLPS is a major discovery in recent cell biology, with LCDs identified as key molecular drivers of this process [5]. LLPS describes the demixing of biomolecules (e.g., proteins, nucleic acids) from a homogeneous solution into concentrated, liquid-like condensates. These condensates, which include nucleoli, stress granules, P-bodies, and transcription factories, are organized by the multivalent interaction networks of LCDs. Within these networks, each LCD-containing molecule can engage in simultaneous, weak interactions with multiple partners, forming a dynamic, “mesh-like” architecture [27,28]. The formation of this interconnected network reduces the system’s free energy, thereby promoting phase separation [29].

The contribution of LCDs to LLPS depends on their specific composition and properties. For instance, tyrosine-rich LCDs typically promote robust phase separation, while glutamine-rich LCDs tend to form more stable, gel-like structures [26]. Furthermore, the length and precise amino acid sequence of an LCD influence the kinetics and thermodynamics of phase separation; longer LCDs generally have a lower critical concentration for condensation than shorter ones [13,28]. LCD-driven LLPS plays a crucial role in diverse biological contexts. Within the nucleus, it facilitates the assembly of membraneless organelles like transcription factories, spliceosomes, and nucleoli, thereby regulating gene expression and RNA processing. In the cytoplasm, LCD-mediated condensation forms stress granules, P-bodies, and signaling complexes, which are essential for mRNA metabolism and signal transduction [30,31]. The involvement of LCDs in cytoskeleton organization and membrane-associated processes further underscores LLPS as a widespread mechanism for intracellular organization [5].

### 3.3. Signal Transmission and Environmental Response

LCDs play a crucial role in cellular signaling and environmental response [3]. Their structural flexibility and sensitivity to environmental changes allow them to act as molecular sensors, detecting signals such as pH, temperature, redox status, and metabolite concentration. Different LCD types exhibit specific sensitivities; for instance, histidine-rich LCDs are particularly sensitive to pH changes. In *Drosophila*, the Orb2 protein contains histidine-rich LCDs that function as a pH-sensitive molecular switch in synaptic plasticity [32]. Similarly, methionine-rich LCDs can sense redox states. In yeast, certain proteins with methionine-rich LCDs undergo functional conformational changes under oxidative stress [3,33].

LCDs can also sense specific small molecules. In *Candida albicans*, serine/threonine-rich LCDs are involved in CO_2_ sensing, regulating fungal morphogenesis and pathogenicity [5]. Furthermore, the phase separation behavior of some LCDs is regulated by nucleotides (e.g., ATP, GTP), polyphosphates, and polyamines, which can act as hydrotropes or otherwise modulate interaction dynamics, thereby linking metabolic state to condensate formation [3]. In signal transduction, LCDs often function through phase separation. For example, during stress response, LCD-containing RNA-binding proteins undergo LLPS to form stress granules, which temporarily sequester untranslated mRNAs [5]. Once stress is relieved, these condensates rapidly dissolve, restoring normal translation. This reversible phase separation allows LCDs to mediate dynamic regulation in signaling pathways.

### 3.4. Transcription Regulation and Gene Expression

LCDs are central to transcriptional regulation and gene expression in eukaryotes. Many transcription factors and co-regulators contain LCDs that facilitate gene transcription by recruiting the transcriptional machinery, promoting chromatin remodeling, and regulating elongation [14,34]. During transcription initiation, LCD-containing transcription factors can form transcriptional condensates via LLPS, enriching essential factors like RNA polymerase II and general transcription factors. A key example is the serine-rich LCD in the C-terminal domain (CTD) of RNA polymerase II, where phosphorylation-dependent changes regulate interactions with transcription and mRNA processing factors, thereby coordinating transcription initiation, elongation, and processing [25].

LCDs also contribute to chromatin remodeling and epigenetic regulation. In the mammalian SWI/SNF chromatin remodeling complex, multiple subunits contain LCDs that interact with LCDs in transcription factors, facilitating chromatin structural changes and gene expression modulation [13]. Additionally, LCDs in certain histone-modifying enzymes (e.g., KMT2D) can form an LLPS microenvironment that stabilizes enzyme complexes and promotes histone modification [17].

Emerging research highlights a critical role for LCDs in enhancer function and super-enhancer formation [35,36]. Super-enhancers are genomic regions densely clustered with enhancer elements that define cell identity and drive disease-related gene expression. LCD-containing transcription factors and coactivators form highly concentrated aggregates at super-enhancers via LLPS, significantly amplifying gene expression. In cancer, for instance, aberrant super-enhancers that drive oncogene overexpression often depend on the phase-separation capacity of their LCD-containing transcription factors [35].

### 3.5. Cytoskeleton Organization and Cellular Structure Maintenance

LCDs are vital for maintaining cytoskeleton organization and cellular structure, particularly within the IFs system [21,37]. As a major cytoskeletal component, IFs provide mechanical support and structural stability. The head and tail domains of IF proteins are typically glycine-rich LCDs, which are critical for filament assembly and function. In vimentin IFs, for example, the head-domain LCD forms the core of the filament, while the tail-domain LCD forms lateral connections between protofilaments, conferring exceptional mechanical strength and elasticity [22,38]. These LCDs also mediate interactions with other cellular structures. For instance, the LCDs of nuclear lamins interact with nuclear membrane proteins and chromatin to help maintain nuclear morphology and function [21]. IFs LCDs can also interact with microtubules and actin filaments, coordinating the entire cytoskeletal network.

Recent studies suggest that IF LCDs contribute to the dynamic regulation of cellular structure through phase separation mechanisms [22]. During cell division, for example, the reorganization of the IF network may involve LCD-mediated phase separation and gelation. This dynamic regulation allows cells to adapt their structure and function flexibly under different physiological conditions.

### 3.6. Nuclear Pore Transport

The selective permeability barrier of the nuclear pore complex (NPC) is largely formed by LCDs rich in phenylalanine-glycine (FG) repeat sequences, which facilitate nucleocytoplasmic transport [39]. The function of this barrier hinges on hydrophobic interactions, particularly those mediated by phenylalanine residues. These interactions enable FG-repeat LCDs to form a cohesive, sieve-like meshwork that constitutes the barrier itself [19,40]. This meshwork is permeable to nuclear transport receptors (NTRs) but excludes inert macromolecules.

The transport mechanism relies on the same hydrophobic principle. NTRs, which often possess hydrophobic patches, interact with the FG repeats to transiently disrupt the local meshwork, allowing for selective passage [41,42]. This interaction facilitates a rapid “slide-and-exchange” mechanism, enabling efficient cargo translocation through the NPC [43]. The critical role of hydrophobicity in this process is further underscored by in vitro studies showing that FG-repeat domains can form hydrogels that mimic the NPC’s permeability properties [44]. Recent quantitative assessments using techniques like scanning electrochemical microscopy have directly confirmed the hydrophobicity of the transport barrier within the NPC [45].

In conclusion, hydrophobic interactions are fundamental to the NPC’s permeability barrier, governing both its structural integrity and its selective transport function. The properties of FG-repeat LCDs enable the rapid and efficient translocation of NTR-bound cargo while maintaining a barrier against non-specific macromolecules, highlighting their central role in cellular regulation.

## 4. LCDs and Diseases

### 4.1. LCD Dysfunction in Neurodegenerative Diseases

Dysfunction of LCDs is implicated in various neurodegenerative diseases, including amyotrophic lateral sclerosis (ALS), frontotemporal dementia (FTD), Alzheimer’s disease (AD), Parkinson’s disease (PD), and Spinocerebellar ataxia (SCA) (Table 1) [5,46,47]. A common pathological feature is the loss of normal dynamic regulation in LCD-containing proteins, leading to aberrant aggregation, neuronal dysfunction, and cell death. In ALS and FTD, LCD dysfunction in RNA-binding proteins is a key pathogenic mechanism. For instance, mutations in the LCDs of FET family proteins (FUS, TAF15, EWS), hnRNPA1/2, TDP-43 are linked to familial forms of ALS and FTD [26,48,49,50,51]. These mutations often alter phase separation properties, favoring the formation of irreversible pathological fibrils over dynamic liquid condensates. The D290V mutation in the hnRNPA2 protein LCD, for example, enhances its phase separation propensity and promotes the formation of more stable gel-like structures, which are a primary source of neurotoxicity [52,53].

These mutations often alter phase separation properties, favoring the formation of irreversible pathological fibrils over dynamic liquid condensates. The D290V mutation in the hnRNPA2 protein LCD, for example, enhances its phase separation propensity and promotes the formation of more stable gel-like structures, which are a primary source of neurotoxicity [52,53]. Proline is crucial for TDP-43 LCD’s phase separation. Mutation of proline (P320) in the core phase separation region causes severe protein aggregation, disrupting normal function. In contrast, proline mutations outside this core have no effect. This aggregation indicates abnormally strong interactions between TDP-43 LCDs, with a greater impact than the pathogenic M337V mutation [48]. Proline’s inability to form hydrogen bonds between peptide main chains helps balance LCD interactions, highlighting the sensitivity of these dynamic structures to main chain interactions.

In AD, LCD dysfunction in the Tau protein is central to the formation of neurofibrillary tangles. Tau is a microtubule-associated protein with a C-terminal LCD rich in proline and serine [54]. In the AD brain, abnormal phosphorylation of Tau induces conformational changes in its LCD, increasing its aggregation tendency and ultimately leading to the formation of paired helical filaments (PHFs) and straight filaments (SFs) [55,56,57]. These aggregates disrupt the neuronal cytoskeleton, contributing to dysfunction and death. Furthermore, in chronic traumatic encephalopathy (CTE) and Guam amyotrophic lateral sclerosis/Parkinson’s dementia complex (ALS/PDC), Tau LCDs assemble into fibrils with a distinct “CTE-fold” structure, different from those in AD [58,59]. This structural specificity suggests that distinct conformational changes in LCDs underlie different neurodegenerative diseases.

### 4.2. LCD Dysfunction in Cancer

LCD dysfunction also plays a significant role in cancer pathogenesis (Table 1) [35]. Oncogenic mechanisms involving LCDs primarily include the following: (1) dysregulation of gene expression due to aberrant function of LCD-containing transcription factors and chromatin remodelers [60]; and (2) disruption of RNA metabolism and splicing by malfunctioning LCD-containing RNA-binding proteins [5].

A classic example is the formation of oncogenic fusion proteins via chromosomal translocations involving LCDs [60]. In Ewing sarcoma, the LCD of the EWSR1 protein fuses to the DNA-binding domain of an ETS transcription factor (e.g., FLI1), forming the EWSR1::FLI1 fusion oncoprotein [61]. The EWSR1 LCD drives the formation of aberrant transcriptional condensates via phase separation, promoting oncogene expression and tumorigenesis. The oncogenic potential of EWSR1::FLI1 is highly dependent on the strength of its LCD-mediated interactions, with both excessively strong and weak interactions impairing its transcriptional activation and transforming ability. Similarly, in acute lymphoblastic leukemia, the hnRNPH1-MEF2D fusion protein’s LCD is critical for leukemogenesis [62]. The hnRNPH1 protein contains two LCDs, and a Y408S mutation in the LC2 domain enhances its phase separation, causing a liquid-to-solid transition in the condensates. This alters interactions with other RNA-binding proteins, potentially driving cancer progression [62]. LCD dysfunction in chromatin regulators is also cancer-relevant. For example, the KMT2D protein uses its two LCDs to form a phase-separated microenvironment that stabilizes its enzymatic complex, facilitating H3K4 monomethylation and gene activation [17]. In pancreatic cancer, loss-of-function mutations in KMT2D’s LCDs reduce H3K4me1 levels, dysregulating key transcription factors like LIFR and KLF4 and thereby influencing tumor progression [17].

### 4.3. LCD Dysfunction in Other Diseases

LCD dysfunction is associated with a spectrum of other disorders, including metabolic diseases, autoimmune conditions, and multisystem proteinopathies (Table 1). In Multisystem Proteinopathy (MSP), mutations in the LCD of the hnRNPA2/B1 protein alter its phase separation, leading to pathological aggregation [63]. The D290V mutation in hnRNPA2, for instance, promotes the formation of stable fibrils, impairing the protein’s RNA-binding and transport functions and causing multi-system pathology, including myopathy and Paget’s disease of bone [64,65]. In prion diseases, the conformational plasticity of the prion protein (PrP) LCDs is central to pathogenesis [66]. The PrP LCDs can undergo a transition from an alpha-helical to a beta-sheet-rich conformation, creating an infectious form that template-misfolds other PrP molecules into amyloid fibrils. This “protein-only” mechanism represents a distinct paradigm of infectious disease. Additionally, mutations in the LCDs of intermediate filament proteins are linked to skin and muscle disorders [21]. Keratin LCDs mutations, for example, cause epidermolysis bullosa by disrupting intermediate filament assembly, compromising tissue mechanical integrity and function.

**Table 1:** Representative LCD-related proteins and their functional associations with diseases.

Protein	LCDs Type	Function	Related Diseases	Mutations/Modifications	Reference
FUS	G/Y-rich	transcriptional regulation	ALS	P525L, R495X	[51,67]
TDP-43	G/N-rich	RNA binding and stress granules	ALS and FTD	P320L, M337V	[50,68]
Tau	P/S-rich	Microtubule stability	AD and CTE	Excessive phosphorylation	[54]
EWSR1	Q/Y-rich	Stable transcriptional activation and LLPS	Ewing sarcoma	EWS: FLI1 fusion	[61]
hnRNPA2	R/D-rich	RNA splicing and transport	MSP and ALS	D290V	[52,53]
hnRNPH1	R/G/G-rich	RNA splicing and transcription	acute lymphoblastic leukemia	Y408S	[62]
KMT2D	Q-rich	Chromatin modification	Pancreatic cancer	LCDs loss mutation	[17]

### 4.4. Therapeutic Targeting of LCDs

Given their central role in pathogenesis, LCDs are emerging as promising therapeutic targets [5]. Strategies aim to modulate LCDs’ phase separation, disrupt aberrant protein interactions, and inhibit pathogenic aggregation. For neurodegenerative diseases, a major focus is on developing small molecules that modulate LCDs’ phase behavior. These include small-molecule crowding agents that shift phase separation equilibrium away from irreversible aggregation, and compounds that bind specifically to LCDs to inhibit abnormal interactions [69]. For instance, small molecules targeting the FUS LCDs can reduce its phase separation propensity and diminish neurotoxic aggregate formation [70].

In oncology, strategies target aberrant transcriptional condensates driven by oncogenic LCDs. Small molecules designed to disrupt the LCD-mediated condensates of the EWSR1::FLI1 fusion protein can inhibit oncogene expression and tumor growth [60,71]. Similarly, modulating the phase separation of LCDs within super-enhancers is a potential avenue to selectively silence oncogenes [35].

For prion diseases, efforts are directed at developing antibodies and small molecules that specifically recognize and clear the pathological PrP conformation or stabilize its native state, thereby preventing misfolding and transmission [66]. For instance, monoclonal antibodies can specifically recognize the pathogenic conformation of PrP, promote its clearance, and prevent disease transmission [72]. In addition, some small-molecule compounds that can stabilize the normal conformation of PrP have also been developed, which can prevent PrP from transforming into a pathogenic conformation [73,74].

## 5. Research Methods and Technological Advances in LCDs

### 5.1. Biochemical and Biophysical Methods

A diverse suite of biochemical and biophysical techniques is employed to study the structural properties and functional mechanisms of LCDs (Table 2). Circular dichroism (CD) and nuclear magnetic resonance (NMR) spectroscopy are commonly used to probe secondary structure and dynamic properties [2]. CD provides information on the relative proportions of secondary structures (e.g., alpha-helices, beta-sheets, random coils), while NMR offers residue-level insights into local environments, dynamics, and intermolecular interactions. For instance, NMR has been used to analyze the dynamic characteristics of the EWS protein LCDs and the role of tyrosine residues in its phase separation [26].

Analytical ultracentrifugation (AUC) is a key method for studying LCDs’ self-association and aggregation [75], enabling precise measurement of molecular weight, oligomeric state, and interaction affinity. In the same study of the EWS LCDs, AUC characterized its self-association behavior and phase separation propensity [26]. Light scattering techniques, including dynamic light scattering (DLS) and static light scattering (SLS), are vital for characterizing the phase separation process and condensate properties [76,77]. DLS measures droplet size and diffusion, while SLS provides information on molecular weight and concentration, together offering insights into the kinetics and thermodynamics of LCDs phase separation.

Fluorescence correlation spectroscopy (FCS) and fluorescence recovery after photobleaching (FRAP) are instrumental methodologies for investigating the dynamic properties of LCDs. Each of these techniques offers distinct advantages in elucidating molecular dynamics across various temporal and spatial dimensions. FCS facilitates the detailed examination of molecular dynamics within cellular environments by analyzing minute fluctuations in fluorescence intensity [78]. This analysis enables the measurement of diffusion coefficients and interaction dynamics of fluorescently labeled LCDs in solution [79]. Conversely, FRAP is employed to assess the diffusion of molecules within cell membranes or other biological structures. By monitoring the recovery of fluorescence signals, FRAP provides insights into molecular mobility and membrane microdomain interactions, thereby allowing the evaluation of the dynamic exchange rates of LCDs in condensed matter, which reflects the fluidity and dynamic characteristics of such systems [80,81]. For instance, in the investigation of the LCDs of ARID1A, ARID1B, and SS18 proteins, FRAP has demonstrated that the aggregates formed by these LCDs exhibit liquid-like properties [13]. FCS and FRAP are potent methodologies for examining the dynamic properties of LCDs, each offering distinct perspectives and information. The integration of these techniques enables researchers to achieve a holistic understanding of molecular dynamics across various temporal and spatial scales, thereby facilitating advanced investigations into the intricate dynamic processes of biomolecules [82].

### 5.2. Structural Biology Methods

Structural biology methods have provided critical insights into the three-dimensional architectures and conformational dynamics of LCDs (Table 2) [2]. While X-ray crystallography faces challenges due to the intrinsic disorder of LCDs, it has successfully resolved structures of some LCDs under specific conditions, such as the beta-sheet structure of the intermediate filament head domain [81]. Nevertheless, researchers have successfully deciphered the crystal structures of some LCDs, such as the β-folding structure of the intermediate silk protein head domain [21].

Cryo-electron microscopy (cryo-EM) has become a premier tool for studying LCDs [68]. Its ability to analyze structures under near-native conditions makes it ideal for capturing dynamic states and the structures of pathological aggregates. For example, cryo-EM has revealed the structures of paired helical and straight filaments formed by Tau protein LCDs in Alzheimer’s disease brains, elucidating their pathological conformational changes [83,84]. Cryo-electron tomography (cryo-ET) combined with subtomogram averaging allows in situ structural analysis of LCDs within complex cellular contexts [22,85]. This approach was used to decipher the complex helical structure of vimentin intermediate filaments, revealing the roles of head and tail LCDs in filament assembly [22]. Hydrogen/deuterium exchange mass spectrometry (HDX-MS) is another powerful method that probes structural dynamics and solvent accessibility by measuring hydrogen exchange rates, providing insights into LCD conformational changes and interactions [2,86]. HDX-MS can measure the hydrogen/deuterium exchange rates in different regions of proteins, reflecting their structural dynamics and solvent accessibility. This method is particularly suitable for studying the dynamic conformational changes in LCDs and their interactions with other molecules.

### 5.3. Cell Biology Methods

Cell biology techniques are essential for studying the function and dynamic behavior of LCDs in their native cellular environment (Table 2). Fluorescence microscopy is the most common method, typically involving the fusion of LCDs to fluorescent proteins (e.g., GFP, mCherry) for real-time observation in live cells. This has been used to study the phase separation and nucleocytoplasmic distribution of the FUS protein LCDs [87,88]. Fluorescence Resonance Energy Transfer (FRET) and Bioluminescence Resonance Energy Transfer (BRET) are versatile techniques applicable to both solution-based in vitro assays and live-cell in vivo studies [89,90]. This discussion underscores their distinctive utility in investigating LCD dynamics within native cellular environments. In live-cell contexts, FRET and BRET facilitate the real-time monitoring of protein–protein interactions or conformational changes in LCD-containing proteins under physiological conditions [91,92]. These interactions are challenging to replicate in reconstituted solution systems. The inclusion of FRET/BRET in this section highlights their specific applicability to cellular studies, thereby complementing solution-based fluorescence techniques, such as FCS, which are addressed in the section on biochemical and biophysical methods. In addition, Protein complementation assays (PCAs), which reconstitute a reporter protein upon interaction of its fused fragments, are particularly effective for detecting weak and transient LCD interactions [93]. This method divides the reporter protein into two inactive fragments and fuses them with potentially interacting proteins. When the two proteins interact, the two fragments of the reporter protein recombine to form an active reporter molecule. This method is particularly suitable for studying the weak and dynamic interaction networks of LCDs.

### 5.4. Computational Biology and Bioinformatics Methods

Computational approaches are indispensable for identifying LCDs and predicting their properties from sequence data (Table 2) [2]. Bioinformatics tools like SEG, functional Liquid–Liquid Phase Separation predictor (fLPS), and LCD-Compass use various algorithms to identify and classify LCDs based on sequence complexity and amino acid composition [3,94]. Molecular dynamics (MD) simulations model the conformational changes and interaction dynamics of LCDs at atomic resolution, providing mechanistic insights. For instance, MD simulations revealed how tyrosine residues mediate self-association and phase separation in the EWS LCDs [26,95]. Coarse-grained models, which simplify molecular details, enable the simulation of large-scale phase separation processes, revealing how amino acid composition influences condensation kinetics and thermodynamics [96]. Machine learning methods are increasingly applied to predict LCD properties (e.g., secondary structure, phase separation propensity) from sequence data and even to design LCDs with specific functional characteristics.

### 5.5. Emerging Technologies and Future Development Directions

Emerging technologies and innovative applications of established methodologies hold the potential to significantly advance research on LCDs. While single-molecule techniques (e.g., smFRET, smFS) and super-resolution microscopy (e.g., STED, STORM, SIM) are commercially available and well-established, their specialized application in the field of LCD research remains an advanced and expanding area [26,97]. These technologies provide a novel perspective by elucidating the dynamic behavior and heterogeneity of LCDs at the molecular level, thereby enhancing our understanding of their functional mechanisms [98,99]. Super-resolution microscopy, by surpassing the diffraction limit of light, enables nanometer-scale visualization of the internal architecture and dynamics of LCD-based condensates within cellular environments. This advanced imaging technique facilitates the investigation of the internal structure and dynamic alterations of condensed matter formed by LCDs in situ [97,100]. For example, super-resolution microscopy can be employed to examine the intricate internal structures and dynamic transformations of LCD-formed condensates within cells.

In addition to the advanced applications of established methodologies, three progressive directions are set to revolutionize the field. Firstly, artificial intelligence (AI), with a particular emphasis on deep learning and protein language models (PLMs), is emerging as a pivotal tool in LCD studies. It extends beyond mere sequence-structure predictions to provide actionable biological insights. AI holds significant potential in predicting phase separation tendencies, designing novel LCDs, facilitating drug discovery, and addressing other critical aspects [101,102]. Secondly, spatial transcriptomics and spatial proteomics are becoming transformative for LCD research by overcoming the limitations of bulk analyses. These techniques enable the mapping of LCD-containing proteins and their RNA targets within tissue-specific contexts, illuminating their roles in development and disease [103]. Thirdly, although single-molecule techniques are well-established in vitro, their application to live organisms through in vivo single-molecule tracking (SMT) represents a burgeoning frontier in LCD research [104,105]. This approach surpasses the constraints of cell culture models, allowing for the direct observation of LCD behavior amidst the complexities of tissue architecture, cellular crowding, and physiological signaling. Such insights are essential for comprehending the physiological and pathological roles of LCDs.

These emerging directions, in conjunction with the advanced application of established methodologies, are poised to address pivotal unresolved questions in LCD research. These include the spatiotemporal regulation of phase separation in vivo, the causal relationship between LCD heterogeneity and disease, and the rational design of therapies targeting LCDs.

**Table 2:** Summary of Key Experimental Methods for Studying LCDs.

Method Category	Specific Technique	Objective/Goal	Example Application
Biochemical & Biophysical	Circular Dichroism (CD)	Characterize secondary structure composition (α-helix, β-sheet, random coil)	Characterizing structural transitions in various LCD types [2]
	Nuclear Magnetic Resonance (NMR)	Residue-level insights into dynamics, local environment, and interactions	Analyzing the role of tyrosine in EWS LCD phase separation [26]
	Analytical Ultracentrifugation (AUC)	Study self-association, oligomeric state, and aggregation	Characterizing self-association of the EWS LCD [26]
	Dynamic/Static Light Scattering (DLS/SLS)	Characterize phase separation kinetics, droplet size, and thermodynamics	Studying condensation initiation of hnRNPA1 LCD [77]
	Fluorescence Recovery After Photobleaching (FRAP)	Assess fluidity and dynamics of molecules within condensates	Demonstrating liquid-like properties of SWI/SNF complex LCDs [13]
Structural Biology	Cryo-Electron Microscopy (Cryo-EM)	Determine high-resolution structures of aggregates and filaments	Solving structures of Tau fibrils from Alzheimer’s brain [83,84]
	Cryo-Electron Tomography (Cryo-ET)	In situ structural analysis of complexes within cells	Revealing the role of vimentin head/tail LCDs in filament assembly [22]
	Hydrogen/Deuterium Exchange MS (HDX-MS)	Probe structural dynamics and solvent accessibility	Studying conformational changes and interactions of LCDs [86]
Cell Biology	Fluorescence Microscopy	Visualize subcellular localization and phase separation in live cells	Observing nucleocytoplasmic distribution and condensation of FUS LCD [88]
	Fluorescence Resonance Energy Transfer (FRET)	Detect direct protein–protein/protein–RNA interactions	US LCD: Validate binding to RNA during phase separation [91,92]
	Protein Complementation Assay (PCA)	Detect weak, transient protein–protein interactions	Mapping the dynamic interaction network of LCDs [93]
	Super-Resolution Microscopy (STED/STORM)	Visualize subcellular localization and fine structure of LCD condensates.	Resolve spatial organization [97]
Computational Biology	Molecular Dynamics (MD) Simulations	Model conformational changes and interaction dynamics at atomic resolution	Revealing how tyrosine residues mediate π–π stacking in EWS LCD [26,95]
	Bioinformatics (e.g., fLPS, LCD-Compass)	Identify LCDs and predict properties from sequence data	Proteome-wide discovery and classification of LCDs [3]

## 6. Conclusions and Prospect

### 6.1. Main Findings of LCDs

Research on LCDs has undergone a paradigm shift, from being perceived as “garbage sequences” to being recognized as critical “functional elements,” reflecting our deepening understanding of proteome complexity and cellular organization [2]. This evolving perspective has led to several pivotal discoveries. Firstly, LCDs serve as a primary source of protein functional diversity. Their distinctive amino acid composition and sequence patterns endow them with a broad array of structural and functional characteristics, particularly their ability to transition dynamically between disordered and ordered states, which confers high structural flexibility. This inherent plasticity allows LCDs to fulfill crucial roles in a variety of biological processes, including protein interactions, phase separation, signal transduction, and transcriptional regulation [12]. Secondly, LCDs are integral to the organization of intracellular space through dynamic regulation. They facilitate the formation of biomolecular condensates via LLPS, thereby promoting the spatial organization of biochemical reactions within membraneless organelles. This LLPS mechanism is now recognized as a fundamental principle of cellular organization [6]. Finally, given their sensitivity to environmental cues, the dysfunction of LCDs is closely linked to various diseases. This sensitivity means that under pathological conditions, alterations in the phase separation behavior and aberrant aggregation of LCDs are key factors in the pathogenesis of neurodegenerative diseases, cancer, and prion diseases. These insights provide a new perspective for understanding disease mechanisms and developing novel therapeutic strategies [5].

### 6.2. Challenges and Limitations of LCD Research

Despite significant progress, the study of LCDs faces considerable challenges. A primary obstacle is their structural dynamics and heterogeneity. LCDs are inherently disordered or form only transient structures in solution, rendering traditional structural biology techniques inadequate for elucidating their complete three-dimensional conformations. Moreover, their structure and function exhibit high sensitivity to environmental conditions such as pH, temperature, ion strength, and protein concentration, which significantly increases experimental complexity [2]. Methodological constraints also constitute a significant barrier. Current techniques possess inherent limitations; for instance, while in vitro studies allow precise control of conditions, they may not replicate the intricate cellular environment, whereas in vivo studies, though physiologically relevant, make it difficult to control variables and dissect molecular mechanisms. Methods for studying LCDs’ phase separation and aggregation also need refinement, particularly in distinguishing physiological condensation from pathological aggregation. Lastly, attributing specific biological functions to LCDs remains a formidable challenge. Although their involvement in numerous cellular processes is acknowledged, the precise mechanisms of action for most individual LCDs are still unclear [5]. This challenge is compounded by the functional interplay between LCDs and other structured domains within a protein, making it difficult to isolate the contribution of the LCDs themselves.

### 6.3. Future Research Directions and Prospects

Research on LCDs is a rapidly advancing field, with future studies anticipated to progress across several critical areas. Firstly, gaining a more profound understanding of the structure-function relationship will be crucial. This includes determining the structural conformations of a broader range of LCDs under various conditions, elucidating how amino acid composition dictates their behavior, and revealing the mechanisms underlying their interactions with other molecules. Secondly, future investigations should aim to elucidate the physiological functions and regulatory mechanisms of LCDs within cells, focusing on their roles across different cell types and conditions, the signaling pathways they modulate, and the precise cellular control of their phase separation. Thirdly, the development of novel therapeutic strategies targeting LCDs presents a promising avenue. This could involve designing small molecules to modulate their phase separation, developing biologics to clear pathological aggregates, and exploring LCD-based biomaterials. Finally, the integration of multi-omics data and systems biology approaches will offer a comprehensive perspective. By combining genomics, transcriptomics, and proteomics data, researchers can systematically analyze LCDs’ functional networks, and computational models can be built to predict their behavior [106].

In short, LCD research is at the forefront of life sciences. Advancements in this field will yield critical insights into fundamental biological principles and novel therapeutic avenues. With continuous methodological improvements and increasingly deep investigation, LCD research is poised to make groundbreaking contributions to life science and medicine.

## Data Availability

No new data were created or analyzed in this study.

## Abbreviations

The following abbreviations are used in this manuscript:

LCDs	Low-complexity domains
LLPS	Liquid–liquid phase separation
ALS	Amyotrophic lateral sclerosis
FTD	Frontotemporal dementia
IFs	Intermediate filaments
cryo-EM	Cryo-electron microscopy
cryo-ET	Cryo-electron tomography
CD	Circular dichroism
NMR	Nuclear magnetic resonance
AUC	Analytical ultracentrifugation
DLS	Dynamic light scattering
SLS	Static light scattering
FCS	Fluorescence correlation spectroscopy
FRAP	Fluorescence recovery after photobleaching
FRET	Fluorescence resonance energy transfer
BRET	Bioluminescence resonance energy transfer
MD	Molecular dynamics
STED	Stimulated emission depletion microscopy
STORM	Stochastic optical reconstruction microscopy
SIM	Structured illumination microscopy
fLPS	functional Liquid–Liquid Phase Separation predictor
AD	Alzheimer’s disease
PD	Parkinson’s disease
PHFs	Paired helical filaments
SFs	Straight filaments
CTE	Chronic traumatic encephalopathy
MSP	Multisystem proteinopathy
HDX-MS	Hydrogen/deuterium exchange mass spectrometry
PrP	Prion protein
KMT2D	Lysine methyltransferase 2D
FET	FUS/EWS/TAF15
SWI/SNF	Switch/sucrose non-fermentable
